# Shedding Light on Animal Cryptochromes

**DOI:** 10.1371/journal.pbio.0060168

**Published:** 2008-07-01

**Authors:** Kira E O'Day

Anyone who's neglected a houseplant for any length of time knows that plants can't survive without light. But it's more complicated than that; in addition to serving as an energy source, light is used by plants as a signal to sense and respond to the environment. For example, both red and blue light send the signal for maturation and flower and seed development. Depriving a plant of such light signals disrupts a variety of growth processes such as early seedling development, leaf and stem expansion, and initiation of flowering, as well as circadian clock entrainment. Cryptochromes are photoreceptor proteins used by plants to mediate the effects of blue light; they are also found in animals like flies and mice, where they help to regulate the circadian clock. In plants, exposure to blue light results in the reduction (gain of electrons) of flavin pigments that are bound by the cryptochrome. Blue light activates plant cryptochromes by reducing the flavins, but researchers weren't sure exactly how blue light activates fly cryptochromes, or if mammalian cryptochromes even respond to light at all.

In a new study, Nathalie Hoang et al. set out to determine how animal cryptochromes respond to blue light, and if mammalian cryptochromes are capable of blue light activation. The researchers performed a series of experiments to measure the effects of blue light irradiation on cryptochromes in living flies and in insect cell lines (Sf21) expressing high levels of fly or human cryptochrome (Dmcry and Hscry1, respectively). They found that not only is the bound flavin molecule in an intermediately reduced, unpaired electron (radical) state in activated animal cryptochromes, similar to what is seen in plants, but that mammalian cryptochrome is fully capable of responding to light in living animals.

Although you might think that animal cryptochromes are activated the same way that plant cryptochromes are, in fact, plant and animal cryptochromes evolved independently from different DNA photolyase proteins, which rely on energy from blue light to repair DNA damaged by ultraviolet radiation. Like plant cryptochromes, DNA photolyases have a bound flavin molecule. However, in plant cryptochromes, flavin goes from a fully oxidized resting state (able to receive electrons) to a radical intermediate state when activated by blue light, whereas flavins bound by DNA photolyases are generally completely reduced.

**Figure:**
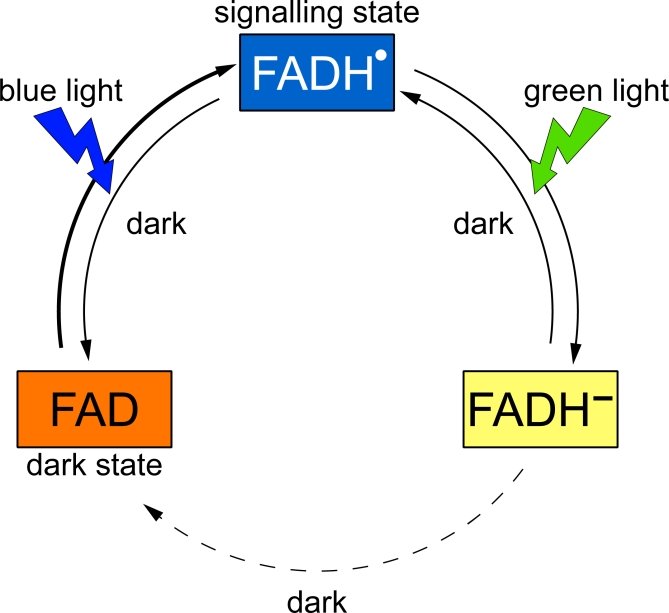
Using biophysical techniques to monitor cryptochrome blue light photoreceptors in living cells as they undergo a chemical redox reaction in response to light, researchers show that cryptochromes share similar properties in organisms ranging from plants to higher animals and humans.

After first confirming that flavin was indeed the photoreactive pigment in living flies and in the cell lines, and that it is fully oxidized in the resting state (as in plant cryptochromes), the researchers sought to more fully characterize what was happening to animal cryptochromes in living cells irradiated with blue light. To do this, they exposed Sf21 cells to blue light and then measured changes in levels of oxidized flavins based on a decrease in absorption at 450 nm, the wavelength at which protein-bound oxidized flavins absorb the most energy. They found that in both Dmcry- and Hscry1-expressing cells, excitation progressively decreased over time, indicating that the crytochrome-bound flavin molecules were being reduced.

To determine whether animal cryptochrome was being reduced to a radical intermediate state, as in plant cryptochromes, or to a fully reduced state, as in DNA photolyases, the researchers performed whole-cell electron paramagnetic resonance (EPR) spectroscopy (a technique used to detect radicals) on the Sf21 cells. They found that a radical accumulated in the cryptochrome-expressing cells after irradiation with blue light that was not found in control, non–cryptochrome expressing Sf21 cells, indicating that the activation of both Dmcry and Hscry involves light-dependent flavin reduction and accumulation of active, radical cryptochrome.

In plant cryptochromes, the biologically active signaling state induced by blue light is reversed by green light, which converts the active radical to a fully reduced, inactive form, which then reoxidizes in the dark to the original resting state, completing the photocycle. To confirm that the photoconversion they had witnessed was biologically relevant in a living animal, the researchers exposed living flies and Sf21 cells to blue light only or to a blue light–green light combination. By measuring protein degradation levels in the flies and changes in light absorbance at 450 nm in the Sf21 cells, they confirmed that green light acted as an antagonist to blue light activation and that Dmcry was capable of completing the photocycle in vivo.

Although the role of Dmcry as a light-sensing input for the circadian clock is well established, the same can't be said for mammalian cryptochromes. Mouse cryptochromes (Mcry1 and Mcry2) interact with key molecular components of the circadian clock; knocking out both *Mcry1* and *Mcry2* results in a complete loss of circadian rhythms for behaviors such as wheel running. However, these functions are light independent; circadian rhythms are perturbed in *Mcry1*- and *Mcry2*-knockout mice regardless of exposure to light. Other than a decrease in pupil response in *Mcry* knockouts, a functional role for light in mammalian cryptochrome activation has not been demonstrated. To find out if mammalian cryptochrome is capable of being activated by light in a living organism, the researchers created a line of Hscry1-expressing flies. When these flies were irradiated with full-spectrum white light, there was a significant decrease in Hscry protein levels, indicating that Hscry1 is activated by light and undergoes light-dependent proteolysis in much the same way as native Dmcry protein in living flies.

Having determined that animal cryptochrome photocycles are similar to those found in plants and that mammalian cryptochromes are fully capable of reacting to light, what's next? By showing that Hscry1 responds to light in a living organism, Hoang et al. have provided an excellent reason to step up the search for light-dependent functions of mammalian cryptochromes. Since Hscry1 is expressed in many different tissues, some of which, like the retina, are close to the surface of the body, Hscry1 could easily be activated by light. Thus, mammalian cryptochromes may have as-yet undiscovered light-activated novel biological functions, perhaps for nonvisual light detection, where it might operate in signaling pathways to cause subtle changes in protein expression levels or behavior in response to light exposure.

